# Supplementary material for: Extensive characterization of a Williams syndrome murine model shows Gtf2ird1‐mediated rescue of select sensorimotor tasks, but no effect on enhanced social behavior
Source: Genes Brain Behav. 2023 Jun 27;22(4):e12853. doi: 10.1111/gbb.12853 (PMC10393419; doi:10.1111/gbb.12853)
Supplement: Supplementary file 1 — TABLE S1. Statistical information for Figure 1 – Molecular Validation. TABLE S2. Statistical information for Figure 2 – Sensorimotor Tasks. TABLE S3. Statistical information for Figures 3 and 4 – Anxiety and Fear‐Related Tasks, and Developmental Assessment. TABLE S4. Statistical information for Figures 5 and 6 – Social Behavior Tasks and Center Avoidance Assessment. [file GBB-22-e12853-s001.pdf]

Supplemental Information

Extensive characterization of a Williams Syndrome murine model shows *Gtf2ird1*-mediated rescue of select sensorimotor tasks, but no effect on enhanced social behavior

Kayla R. Nygaard, Susan E. Maloney, Raylynn G. Swift, Katherine B. McCullough, Rachael E. Wagner, Stuart B. Fass, Krassimira Garbett, Karoly Mirnics, Jeremy Veenstra-VanderWeele
Joseph D. Dougherty

Table S1. Statistical information for Figure 1 – Molecular Validation

Figure		Method	Target	Genotype	n	Test	BY	Results	Pairwise Comparisons
1	B	qPCR	Gtf2ird1	WT	5	t-test	Geno	t=-5.247, p=0.000776	N/A
				TG	5				
	C	Western	Gtf2ird1	WT	3	t-test	Geno	t=-1.991, p=0.048	N/A
				TG	5				
	E	qPCR	Gtf2ird1	WT	6	ANOVA	Geno	F(3,20)=22.190, p=0.000001	WT - TG: 0.000107
				TG	6				WT - CD: 0.004
				CD	6				WT - TG/CD: 0.711
				TG/CD	6				CD - TG/CD: 0.001
	F	qPCR	Gtf2i	WT	6	ANOVA	Geno	F(3,20)=12.818, p=0.000068	WT - TG: 0.067
				TG	6				WT - CD: 0.01
				CD	6				WT - TG/CD: 0.002
				TG/CD	6				CD - TG/CD: 0.547
	H	Western	Gtf2ird1	WT	3	ANOVA	Geno	F(3,8)=9.918, p=0.005	WT - TG: 0.061
				TG	3				WT - CD: 0.016
				CD	3				WT - TG/CD: 0.388
				TG/CD	3				CD - TG/CD: 0.004

Table S2. Statistical information for Figure 2 – Sensorimotor Tasks

Figure		Task	Variable	Geno	n	Test	BY	Results
2	C	Open Field	Distance Travelled	WT	29	ANOVA	CD x TG x Sex	TG: F(1,86)=3.639, p=0.06
				TG	21			CD: F(1,86)=1.648, p=0.203
				CD	20			Sex: F(1,86)=1.974, p=0.164
				TG/CD	24			TG*CD: F(1,86)=0.059, p=0.809
	E	Sensorimotor Battery: Walk	Time to Leave Square	WT	20	Kruskal-Wallis	Geno/ Sex	H(7)=3.735, p = 0.810
				TG	21			
				CD	17			
				TG/CD	19			
	G	Sensorimotor Battery: Inverted Screen	Time on Screen	WT	20	Kruskal-Wallis	Geno	H(3)=30.208, p=0.000001<br>CD < WT p=0.00002<br>CD < TG p<0.001<br>TG/CD < WT p=0.002<br>TG/CD < TG p<0.001
				TG	21			
				CD	17			
				TG/CD	19			
	I	Sensorimotor Battery: Pole	Time on Pole	WT	20	Kruskal-Wallis	Geno	H(3)=16.709, p=0.000811<br>CD < TG p=0.002<br>CD < WT p=0.00036<br>TG/CD < TG p=0.05<br>TG/CD < WT p=0.014
				TG	21			
				CD	17			
				TG/CD	19			
	J	Sensorimotor Battery: Ledge	Average Success	WT	20	Kruskal-Wallis	Geno	H(3)=29.487, p=0.000002<br>CD < TG p=0.0004<br>CD < WT p=0.033<br>TG/CD < TG p=0.0000006<br>TG/CD < WT p=0.0004
				TG	21			
				CD	17			
				TG/CD	19			
	L	Sensorimotor Battery: Platform	Average Success	WT	20	Kruskal-Wallis	Geno	H(3)=16.919, p=0.000734<br>CD < TG/CD p=0.046<br>CD < TG p=0.001<br>CD < WT p=0.00012
				TG	21			
				CD	17			
				TG/CD	19			
	N/O	Rotarod: Accelerating	Latency to Fall	WT	20	rmANOVA	CD x TG x Sex	TG: F(1,69)=1.525, p=0.221
				TG	21			CD: F(1,69)=35.227, p=1.06x10-7
				CD	17			Sex*CD*TG: F(1,69)=4.461, p=0.038
				TG/CD	19			TG*CD: F(1,69)=6.977, p=0.01
	Q	Marble Burying	Marbles Buried	WT	29	Kruskal-Wallis	Geno	H(3)=34.458, p=1.586x10^{-7}<br>CD < TG & WT p=0.0003<br>TG/CD < TG & WT p=0.00003
				TG	21			
				CD	20			
				TG/CD	24			
	R	Marble Burying	Distance Travelled	WT	29	ANOVA	CD x TG x Sex	TG: F(1,89)=0.017, p=0.895
				TG	21			CD: F(1,89)=36.953, p=3.0x10^{-8}
				CD	20			Sex: F(1,89)=15.50, p=0.0002
				TG/CD	24			TG*CD: F(1,89)=1.560, p=0.215
	T	Acoustic Startle/PPI	Weight	WT	15	ANCOVA	CD x TG x Sex with Weight Covariate	TG: F(1,47)=0.854, p=0.360
				TG	16			CD: F(1,47)=21.429, p=0.00003
				CD	11			Sex: F(1,47)=188.1, p=4.8x10^{-18}
				TG/CD	12			TG*CD: F(1,47)=0.414, p=0.523

	U	Acoustic Startle/PPI	Average Startle to 120 dB	WT	15	ANCOVA	CD x TG x Sex with Weight Covariate	TG: F(1,46)=12.5217, p=0.0009
				TG	16			CD: F(1,46)=0.119, p=0.731
				CD	11			Sex: F(1,46)=1.336, p=0.254
				TG/CD	12			TG*CD: F(1,46)=0.430, p=0.515
	V	Acoustic Startle/PPI	Average Startle Across Sound Pressure Levels	WT	15	ANCOVA	CD x TG x Sex with Weight Covariate	TG: F(1,46)=18.90, p=0.00008
				TG	16			CD: F(1,46)=0.740, p=0.394
				CD	11			Sex: F(1,46)=0.257, p=0.615
				TG/CD	12			TG*CD: F(1,46)=0.742, p=0.394
	W	Acoustic Startle/PPI	Average Percent Inhibition of Startle	WT	15	ANCOVA	CD x TG x Sex with Weight Covariate	TG: F(1,46)=0.659, p=0.421
				TG	16			CD: F(1,46)=2.608, p=0.113
				CD	11			Sex: F(1,46)=1.437, p=0.237
				TG/CD	12			TG*CD: F(1,46)=4.772, p=0.034

Table S3. Statistical information for Figures 3 and 4 – Anxiety and Fear-Related Tasks, and Developmental Assessment

Figure		Task	Variable	Geno	n	Test	BY	Results
3	B/C	Open Field	Percent Time in Center	WT	29	ANOVA	CD x TG x Sex	TG: F(1,86)=6.661, p=0.012
				TG	21			CD: F(1,86)=11.930, p=0.0009
				CD	20			Sex: F(1,86)=13.276, p=0.0005
				TG/CD	24			TG*CD: F(1,86)=0.256, p=0.614
	E	Elevated Plus Maze	Percent Time in Open Arms	WT	29	ANOVA	CD x TG x Sex	TG: F(1,86)=0.686, p=0.41
				TG	21			CD: F(1,86)=0.03, p=0.864
				CD	20			Sex: F(1,86)=0.986, p=0.323
				TG/CD	24			TG*CD: F(1,86)=0.462, p=0.498
	G	Light/Dark Box	Percent Time in Light Side	WT	20	ANOVA	CD x TG x Sex	TG: F(1,72)=0.570, p=0.453
				TG	21			CD: F(1,72)=1.070, p=0.304
				CD	17			Sex: F(1,72)=0.013, p=0.908
				TG/CD	19			TG*CD: F(1,72)=5.250, p=0.025
	H	Conditioned Fear	Percent Time Freezing - Training	WT	29	rmANOVA	CD x TG x Sex	TG: F(1,85)=2.044, p=0.156
				TG	21			CD: F(1,85)=1.477, p=0.228
				CD	19			Sex: F(1,85)=5.606, p=0.02
				TG/CD	24			TG*CD: F(1,85)=2.914, p=0.091
	I	Conditioned Fear	Percent Time Freezing - Context	WT	29	rmANOVA	CD x TG x Sex	TG: F(1,85)=2.343, p=0.130
				TG	21			CD: F(1,85)=2.644, p=0.108
				CD	19			Sex: F(1,85)=5.650, p=0.02
				TG/CD	24			TG*CD: F(1,85)=0.095, p=0.759
	J/K	Conditioned Fear	Percent Time Freezing - Cue	WT	29	rmANOVA	CD x TG x Sex	TG: F(1,85)=28.497, p=7.7x10^{-7}
				TG	21			CD: F(1,85)=1.214, p=0.274
				CD	19			Sex*TG: F(1,85)=11.876, p=0.0009
				TG/CD	24			TG*CD: F(1,85)=1.628, p=0.205
	Not shown	Shock Sensitivity	mA at Flinch	WT	29	Kruskal-Wallis	Geno	Geno: H(3)=6.885, p=0.076
				TG	21			
				CD	19			
				TG/CD	24			
	C	Maternal Isolation-Induced USVs	Number of Calls	WT	30	rmANOVA	Geno x Sex x Age	Geno: F(3,83)=7.635, p=0.0001
				TG	15			Sex: F(1,83)=3.062, p=0.084
				CD	24			Age: F(2,166)=4.549, p=0.012
				TG/CD	22			Geno*Sex: F(3,83)=0.739, p=0.504
	D	Maternal Isolation-Induced USVs	Call Duration	WT	30	Linear Mixed Model	Geno x Age	Geno: F(3,91)=3.411, p=0.020
				TG	15			
				CD	24			Geno*Age: F(8,142)=1.296, p=0.250
				TG/CD	22			
	E	Maternal Isolation-Induced USVs	Pause Time	WT	30	Linear Mixed Model	Geno x Age	Geno: F(3,89)=2.660, p=0.053
				TG	15			
				CD	24			Geno*Age: F(8,139)=7.009, p=9.4x10^{-8}
				TG/CD	22			P7: F(3,203)=3.332, p=0.021

4	F	Maternal Isolation-Induced USVs	Average Call Frequency	WT	30	Linear Mixed Model	Geno x Age	Geno: $F(3,85)=4.736$, $p=0.004$<br>Geno*Age: $F(8,128)=4.736$, $p=0.00002$<br>P5: $F(3,163)=3.056$, $p=0.030$<br>P7: $F(3,161)=6.728$, $p=0.0003$
				TG	15			
				CD	24			
				TG/CD	22			
	G	Maternal Isolation-Induced USVs	Frequency Range	WT	30	Linear Mixed Model	Geno x Age	Geno: $F(3,96)=1.626$, $p=0.188$<br>Geno*Age: $F(8,144)=6.211$, $p=6.9\times10^{-7}$<br>P7: $F(3,201)=2.974$, $p=0.033$
				TG	15			
				CD	24			
				TG/CD	22			
	H	Maternal Isolation-Induced USVs	Sound Pressure Level	WT	30	Linear Mixed Model	Geno x Age	Geno: $F(3,83)=0.429$, $p=0.773$<br>Geno*Age: $F(8,131)=2.742$, $p=0.008$<br>P5: $F(3,189)=2.733$, $p=0.045$
				TG	15			
				CD	24			
				TG/CD	22			
	F	Maternal Isolation-Induced USVs	Weight at Recording	WT	30	rmANOVA	Geno x Sex x Age	Geno: $F(3,79)=13.691$, $p=2.8\times10^{-7}$<br>Sex: $F(1,79)=0.082$, $p=0.775$<br>Geno*Sex: $F(3,79)=0.816$, $p=0.489$<br>Geno*Age: $F(6,158)=7.558$, $p=3.9\times10^{-7}$
				TG	15			
				CD	24			
				TG/CD	22			
	G	Maternal Isolation-Induced USVs	Temperature at Recording	WT	30	rmANOVA	Geno x Age	Geno: $F(3,87)=2.415$, $p=0.072$<br>Geno*Age: $F(6,174)=0.702$, $p=0.649$
				TG	15			
				CD	24			
				TG/CD	22			
	H	Righting Reflex	Latency to Right	WT	18	Kruskal-Wallis	Geno	Geno: $H(3)=0.595$, $p=0.898$
				TG	14			
				CD	17			
				TG/CD	18			

Table S4. Statistical information for Figures 5 and 6 – Social Behavior Tasks and Center Avoidance Assessment

Figure		Task	Variable	Geno	n	Test	BY	Results
5	B	Tube Test	TTDay 1 Wins Ratio	WT	20	Kruskal-Wallis	Geno/ Sex	H(7)=11.102, p=0.134
				TG	21			
				CD	17			
				TG/CD	19			
	C	Resident Intruder	Average Attacks by Resident	WT	11	ANOVA	CD x TG	TG: F(1,32)=0.030, p=0.864
				TG	7			CD: F(1,32)=1.167, p=0.288
				CD	8			
				TG/CD	10			TG*CD: F(1,32)=0.202, p=0.656
	E	Open Field Social Approach	Percent Social Investigation Time	WT	29	Kruskal-Wallis	Geno	H(3)=8.916, p=0.03<br>WT < TG: p=0.039<br>WT < CD: p=0.013<br>WT < TG/CD p=0.016
				TG	21			
				CD	20			
				TG/CD	24			
	F	Open Field Social Approach	Percent Novel Investigation Time	WT	29	Kruskal-Wallis	Geno	H(3)=13.160, p=0.004<br>WT < TG: p=0.009<br>WT < CD: p=0.014<br>WT < TG/CD p=0.001
				TG	21			
				CD	20			
				TG/CD	24			
	G	Open Field Social Approach	Mean Social Investigation Bout Time	WT	29	Kruskal-Wallis	Geno	H(3)=12.574, p=0.006<br>WT < CD: p=0.001<br>WT < TG/CD p=0.06
				TG	21			
				CD	20			
				TG/CD	24			
	I/J	Open Field Novel Object	Percent Time in Investigation Zone	WT	29	ANOVA	CD x TG x Sex	TG: F(1,86)=2.615, p=0.110
				TG	21			CD: F(1,86)=4.203, p=0.043
				CD	20			Sex: F(1,86)=11.069, p=0.0012
				TG/CD	24			TG*CD: F(1,86)=0.008, p=0.931
	K	Open Field Novel Object	Mean Investigation Bout (s)	WT	29	Kruskal-Wallis	Geno	H(3)=11.271, p=0.010<br>CD > TG p=0.032<br>CD > WT p=0.022<br>TG/CD > TG p=0.016<br>TG/CD >WT p=0.010
				TG	21			
				CD	20			
				TG/CD	24			
	L/N	3-Chamber Social Approach	Social Cup Preference Index	WT	19	ANOVA	CD x TG x Sex	TG: F(1,66)=0.34, p=0.853
				TG	21			CD: F(1,66)=4.99, p=0.029
				CD	15			Sex: F(1,66)=5.414, p=0.023
				TG/CD	19			TG*CD: F(1,66)=0.071, p=0.790
	not shown	3-Chamber Social Approach	Novel Cup Preference Index	WT	19	ANOVA	CD x TG x Sex	TG: F(1,66)=2.694, p=0.105
				TG	21			CD: F(1,66)=3.035, p=0.087
				CD	15			Sex: F(1,66)=1.986, p=0.163
				TG/CD	19			TG*CD: F(1,66)=0.181, p=0.672
	P	Social Operant	Total Rewards (FR1 Mean)	WT	19	ANOVA	CD x TG x Sex	TG: F(1,47)=1.812, p=0.185
				TG	13			CD: F(1,47)=14.07, p=0.0005
				CD	13			Sex: F(1,47)=0.372, p=0.545
				TG/CD	10			TG*CD: F(1,47)=2.496, p=0.121

	Q	Social Operant	Total Rewards (FR3)	WT	18	ANOVA	CD x TG x Sex	TG: F(1,44)=0.228, p=0.635
				TG	12			CD: F(1,44)=1.708, p=0.198
				CD	13			Sex*CD: F(1,44)=4.932, p=0.032
				TG/CD	9			TG*CD: F(1,44)=0.565, p=0.456
	R	Social Operant	Breakpoint (PR)	WT	18	Kruskal-Wallis	Geno	Females: H(3)=0.274, p=0.965
				TG	12			
				CD	13	Kruskal-Wallis	Geno	Males: H(3)=8.092, p=0.044
				TG/CD	9			
6	C	Open Field	% Time in Center	WT	29	ANOVA	CD x TG x Sex	TG: F(1,86)=6.661, p=0.012
				TG	21			CD: F(1,86)=11.930, p=0.0009
				CD	20			Sex: F(1,86)=13.276, p=0.0005
				TG/CD	24			TG*CD: F(1,86)=0.256, p=0.614
	E	Open Field Novel Object	% Time in Center	WT	29	ANOVA	CD x TG x Sex	TG: F(1,86)=0.084, p=0.772
				TG	21			CD: F(1,86)=6.070, p=0.016
				CD	20			Sex: F(1,86)=34.484, $p=7.9x10^{-8}$
				TG/CD	24			TG*CD: F(1,86)=0.211, p=0.647
	G	Marble Burying	% Time in Center	WT	29	ANOVA	CD x TG x Sex	TG: F(1,86)=0.482, p=0.490
				TG	21			CD: F(1,86)=19.826, p=0.00003
				CD	20			Sex: F(1,86)=2.668, p=0.106
				TG/CD	24			TG*CD: F(1,86)=1.495, p=0.225
	I	Open Field Social Approach	% Time in Center	WT	28	ANOVA	CD x TG x Sex	TG: F(1,85)=0.650, p=0.423
				TG	21			CD: F(1,85)=3.336, p=0.071
				CD	20			Sex: F(1,85)=0.862, p=0.356
				TG/CD	24			TG*CD: F(1,85)=1.945, p=0.167
